# Treatment of Horizontal Canal Benign Paroxysmal Positional Vertigo: A New Rehabilitation Technique

**DOI:** 10.1100/2012/160475

**Published:** 2012-04-19

**Authors:** D. Testa, G. Castaldo, C. De Santis, A. Trusio, G. Motta

**Affiliations:** Department of Otorhinolaryngology-ENT Surgery, Second University of Naples, Italy

## Abstract

The aim of this study was to evaluate the effectiveness of a new technical variant applied to the Gufoni's manoeuvre, in the treatment of horizontal canal benign paroxysmal positional vertigo (HSC-BPPV). 87 patients with BPPV of HSC (55 women and 32 men), aged between 21 and 80 years, were randomized either to modified Gufoni's manoeuvre or to the Gufoni's manoeuvre. 93% of patients treated with modified Gufoni's manoeuvre was cured after the first treatment session, of which only 2% had a conversion into PSC-BPPV, while the Gufoni's manoeuvre led to a symptoms resolution in 88% of cases, of which 16% had a conversion into PSC-BPPV. Therefore, the modified Gufoni's manoeuvre shows the same effectiveness in the resolution of symptoms of Gufoni's manoeuvre, but it appears more effective than the latter to reduce the percentage of conversion of the HSC-BPPV into PSC-BPPV (*χ* ^2^ = 6.13, *P* = 0.047).

## 1. Introduction

Benign paroxysmal positional vertigo of horizontal semicircular canal (HSC-BPPV) is a common vestibular disorder, due to the presence of otoconial debris of the utricle inside the endolymph of the posterior or anterior arm of horizontal semicircular canal.

The main symptoms are represented by recurrent brief and sudden episodes of positional vertigo with nausea and vomit, evoked by turning the head from the supine to the lateral position. The nystagmus typically has a latency of few seconds, is paroxysmal, positional, and purely horizontal (geotropic or apogeotropic) that strongly changes in head position. In geotropic forms, the otoliths are located in the nonampullary (or posterior) arm of the pathological horizontal canal; in apogeotropic forms, the otoliths are found in the ampullary (or anterior) arm. The pathological side is usually indicated by nystagmus intensity: the more intense positional nystagmus beats toward the affected ear [1].

BPPV due to canalolithiasis of the horizontal semicircular canal was described the in literature for the first time in 1985, by Cipparone et al. [2] from Italy and McClure [3] from Canada.

In 1989, Pagnini et al. [4] reported 15 cases of HSC-BPPV, hypothesizing that the endolymphatic current, induced by the floating of otoconial debris on the posterior or anterior arm of the HSC, causes, respectively, geotropic or apogeotropic nystagmus.

After few years, many therapeutic approaches have been suggested for HSC-BPPV.

**Figure 1:**
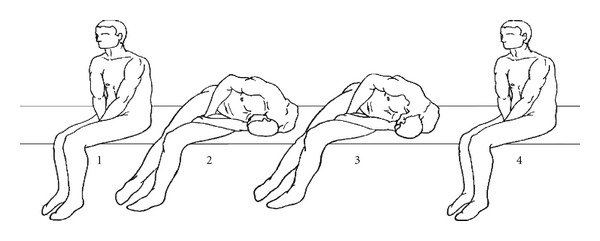
Gufoni's manoeuvre for the geotropic form of right HSC-BPPV [11].

In 1993, Baloh et al. [5] proposed a repositioning manoeuvre, later modified by Lempert [6], based on the rationale that the otoconial debris is moved from the lateral semicircular canal to the utricle, applying an overall brisk rotation of 270° to the patient's head to the healthy side in the supine position, in three steps of 90° each. In 1994, this manoeuvre is modified by Baloh [7] into the Barbecue rotation of 360° assuming that a wider rotation might displace the debris more effectively. In 1994, Vannucchi et al. [8] suggested a rehabilitation technique, “Forced Prolonged Position”, which consists in forcing the patient to remain immobile on the healthy side, for at least 12 hours. In 1998, Gufoni and Mastrosimone [9] proposed a new technique: the patient is briskly tilted, from a seated position, on one side—on the healthy side in the geotropic form and on the impaired side in the apogeotropic form—the head is then turned 45° downwards and held for three minutes; finally, the patient returns to sitting position (Figure 1). In 1999, Libonati and Gufoni [10] described a variation to the Barbecue Rotation Manoeuvre: lying in supine position, the patient's head is briskly turned 90° towards the healthy side, then, while keeping the head turned, the patient is returned to the seated upright position and his head is slowly brought back in axis with the body; finally, he is returned to the supine position.

The Gufoni's manoeuvre is authors' method of choice for the treatment of HSC-BPPV, since it offers significant advantages: it is simple to perform; there are not many movements to execute; it has good tolerability and a high percentage of resolution. However, in a few cases, a conversion of HSC-BPPV into BPPV of posterior semicircular canal (PSC) during the treatment has been observed [12, 13].

The aim of this study was to evaluate the effectiveness of a new technical variation applied to the Gufoni's manoeuvre, in the treatment of BPPV of HSC, in terms of a rapid and complete symptomatic resolution and above all for its ability to reduce the incidence of HSC-BPPV/PSC-BPPV conversion.

## 2. Materials and Methods

From March 2004 to May 2011, in the Department of Otorhinolaryngology of the Second University of Naples, 87 patients with BPPV of HSC (55 women and 32 men), aged between 21 and 80 years (average age 53,5), have been observed; in 50 cases the right HSC was impaired while in 37 the impaired was the left HSC.

All patients underwent a neuro-otological examination, posture evaluation, pure tone audiometric test, timpanometric and stapedial reflex study. The positional nystagmus was evaluated with videonystagmography (VNG) by Dix-Hallpike's manoeuvre and by Pagnini's manoeuvre [14]—patient in supine position and quick rotation of the head to right and to left—to identify the involved semicircular canal. No patient used vestibular suppressant drugs in the last 3 months.

The patients were divided into two randomized groups.

**Group A**

44 patients of whom 32 with geotropic form and 12 with apogeotropic form, treated with the modified Gufoni's manoeuvre.

**Group B**

43 patients of whom 31 with geotropic form and 12 with apogeotropic form, treated with the Gufoni's manoeuvre.

**Figure 2:**
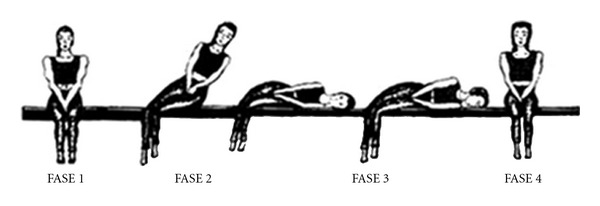
Modified Gufoni's manoeuvre for the geotropic form of right HSC-BPPV.

Modified Gufoni's manoeuvre consists of the following steps (Figure 2).

**Phase 1**

The patient sits upright on the examination table.

**Phase 2**

The patient is tilted on one side—on the healthy side in geotropic form and on the impaired side in apogeotropic form—with a lateral movement of the torso made in two steps: first step consisting in the rapid excursion of 45° and the maintenance of this position for 15 seconds; the second step consisting in the slow completion of the excursion (45°).

**Phase 3**

Patient's head is turned 45° downwards and maintained in this position for 2-3 minutes.

**Phase 4**

The patient is returned to sitting position.

The liberatory manoeuvres have been always carried out under VNG control and repeated at least 3 times, then the patients have been revaluated. The presence of vertigo, or persistence of nystagmus, implies the need for a repetition of the manoeuvre. The patients with conversion to PSC-BPPV, during the treatment, were treated with Semont's manoeuvre: the patient seated with legs outside the examining table and the head turned horizontally 45° to the unaffected ear, quickly laid down, on the side of the affected ear, and this position was maintained for a few minutes. From this critical position, he/she was rapidly turned on the opposite side, maintaining the head rotated. This position was also maintained for a few minutes. Finally, the patient was very slowly sat up.

After the first treatment session, all patients were followed up after 7, 15, and 30 days, thus confirming the resolution of the disorder.

### 2.1. Statistical Methods

The Chi-Square test was used for statistical analysis of the observed data in this study. Results were considered significant at a level of *P* < 0.05 and a 95% confidence interval.

## 3. Results

**Figure 3:**
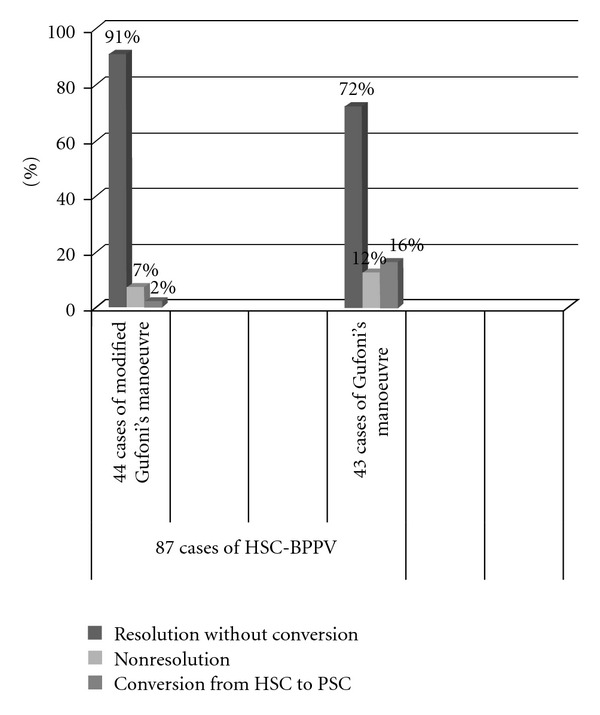
Comparison of results obtained with the 2 rehabilitation techniques: Modified Gufoni's manoeuvre and Gufoni's manoeuvre.

The results obtained are here reported in relation to the different rehabilitation techniques used (Figure 3).

**Group A**

44 cases of HSC-BPPV treated with modified Gufoni's manoeuvre.

- 41 patients (93%) were cured after the first session of which 40 patients (91%) had a complete resolution of symptoms without conversion to PSC-BPPV and 1 patient (2%) had a conversion to PSC-BPPV and was successfully treated with Semont's manoeuvre.
- 3 patients (7%) did not show any benefit after the treatment.

**Group B**

43 cases of HSC-BPPV treated with Gufoni's manoeuvre.

- 38 patients (88%) were cured after the treatment of which 31 patients (72%) had a complete resolution of vertigo and nystagmus without conversion to PSC-BPPV and 7 patients (16%) had a conversion to PSC-BPPV, and were successfully treated with Semont's manoeuvre.
- 5 patients (12%) did not have any benefit after the treatment.

Therefore, the percentage of symptoms resolution after the first session with the modified Gufoni's manoeuvre was not statistically different than those observed with the Gufoni's manoeuvre (93% versus 88%), but the modified Gufoni's manoeuvre appears more effective than Gufoni's manoeuvre (*χ* ^2^ = 6.13, *P* = 0.047) to reduce the percentage of conversion of HSC-BPPV into PSC-BPPV (2% versus 16%).

## 4. Discussion

During the last few years, various theoretically valid therapeutic manoeuvres have been proposed for the treatment of BPPV of HSC. These techniques, aimed to achieve the ampullofugal endocanal progression of the otoconial debris, either by angular accelerations (barbecue rotation techniques), sudden linear accelerations (Gufoni's manoeuvre), slow gravitational sedimentation (Forced Prolonged Position), or with combined methods (Asprella manoeuvre) [11, 15].

Among the techniques proposed, the Gufoni's manoeuvre is authors' method of choice in HSC-BPPV treatment, since it offers significant advantages: it is simple to perform, there are not many movements to execute, the positions are comfortable to the patient, and above all it has a high percentage of success. 

The aim of this manoeuvre is to induce the migration of the otoliths in the utricle through the nonampullary end of the horizontal canal: the quick variation from seated position to lateral decubitus, putting the HSC on a vertical plane, causes the shifting of otoconial debris towards the nonampullary end of the canal; the subsequent 45° downward head rotation moves the otoliths into the utricle. In geotropic forms, the patient is tilted on the healthy side and the otoconial mass is in the posterior arm (non-ampullary) of HSC and floats towards the utricle. In apogeotropic forms, the patient is tilted on impaired side, as the otoliths are in the anterior arm (ampullary) of HSC, and this manoeuvre induces the otoliths migration from the anterior into posterior arm of the horizontal canal. The complete otoliths shifting towards the utricle is effected with the manoeuvre for the geotropic form.

The Gufoni's manoeuvre determines, in a percentage of cases, a conversion from HSC-BPPV into PSC-BPPV, making necessary the use of specific repositioning manoeuvres, such as Semont's manoeuvre. The conversion from HSC-BPPV into PSC-BPPV is due to the otoliths shifting from the nonampullary arm of HSC, through the utricular orifice of the common crus, into nonampullary arm of posterior canal. 

Our technical variation of the Gufoni's manoeuvre is based on the subdivision of the phase 2 of the manoeuvre (lateral movement) in two different steps, the first one consists in a fast and decisive 45°excursion, with the maintenance of the position for 15 seconds; the second step consists in the slow completion of the excursion (45°).

The rationale of this modification is based on the observation that, during phase 2, in the first step of the lateral excursion, the endolymph-otoliths system is moved synergistically with the membranous labyrinth: the sudden head immobilization provokes the immediate stop of the labyrinth, while the endolymph-otoliths system moves to the posterior end of the horizontal canal by inertia. In the second step, the slow excursion induces a coherent, regular and linear flux of the otoconial mass, without turbulence, towards the utricle, far away from the critic area of the common crus.

Our technical variant of the Gufoni's manoeuvre leads to a higher percentage of symptoms resolution: from 88% to 93% after the first session, compared to the Gufoni's manoeuvre, but without statistical significance. Besides, a lower percentage of conversion of the HSC-BPPV into PSC-BPPV has been observed: in particular, the Gufoni's manoeuvre has determined the mentioned conversion in the 16% of cases; with the modified technique, the conversion has been observed only in the 2%.

## 5. Conclusions

This study shows that, in the treatment of BPPV due to HSC canalolithiasis, the modified Gufoni's manoeuvre has the same effectiveness in the resolution of symptoms of Gufoni's manoeuvre, but it appears more effective than the latter to reduce the percentage of conversion of the HSC-BPPV into PSC-BPPV (*χ* ^2^ = 6.13, *P* = 0.047). After the first session of treatment, in fact, we have registered a striking decrease of the incidence of HSC-BPPV/PSC-BPPV conversion (from 16% to 2%) after treatment of patients with the modified Gufoni's manoeuvre.
